# The role of angioembolization in the management of blunt renal injuries: a systematic review

**DOI:** 10.1186/s12894-021-00873-w

**Published:** 2021-08-06

**Authors:** Giovanni Liguori, Giacomo Rebez, Alessandro Larcher, Michele Rizzo, Tommaso Cai, Carlo Trombetta, Andrea Salonia

**Affiliations:** 1 Azienda Sanitaria Universitaria Giuliano-Isontina, Strada di Fiume 447, 34149 Trieste, Italy; 2 Università Vita Salute San Raffaele, Milan, Italy; 3 Ospedale Santa Chiara, Trento, Italy

**Keywords:** Renal, Blunt, Angioembolization, Trauma, Kidney

## Abstract

**Background:**

Recently, renal angioembolization (RAE) has gained an important role in the non-operative management (NOM) of moderate to high-grade blunt renal injuries (BRI), but its use remains heterogeneous. The aim of this review is to examine the current literature on indications and outcomes of angioembolization in BRI.

**Methods:**

We conducted a search of MEDLINE, EMBASE, SCOPUS and Web of Science Databases up to February 2021 in accordance with PRISMA guidelines for studies on BRI treated with RAE. The methodological quality of eligible studies and their risk of bias was assessed using the Newcastle–Ottawa scale

**Results:**

A total of 16 articles that investigated angioembolization of blunt renal injury were included in the study. Overall, 412 patients were included: 8 presented with grade II renal trauma (2%), 97 with grade III renal trauma (23%); 225 with grade IV (55%); and 82 with grade V (20%). RAE was successful in 92% of grade III–IV (294/322) and 76% of grade V (63/82). Regarding haemodynamic status, success rate was achieved in 90% (312/346) of stable patients, but only in 63% (42/66) of unstable patients. The most common indication for RAE was active contrast extravasation in hemodynamic stable patients with grade III or IV BRI.

**Conclusions:**

This is the first review assessing outcomes and indication of angioembolization in blunt renal injuries. The results suggest that outcomes are excellent in hemodynamic stable, moderate to high-grade renal trauma.

**Supplementary Information:**

The online version contains supplementary material available at 10.1186/s12894-021-00873-w.

## Key points

- Angioembolization is effective and safe in stable grade III–IV blunt renal trauma.
- Grade I–II blunt renal injuries need RAE very occasionally.
- A few hemodynamically unstable and or grade V patients might benefit from RAE but the data is not robust enough to support angioembolization as a standard of care in these subgroups.

## Introduction

Renal angioembolization (RAE) is an effective, minimally invasive treatment that was first developed in the 1970s [1]. Renal injuries occur in approximately 10% of all abdominal trauma [2] and the kidney is the third most commonly injured solid organ. Every year, up to 245.000 renal injuries occur worldwide; blunt trauma is responsible for 80–90% of them [2, 3]. Over the last few decades, non-operative management (NOM), including observation, transfusion, bed rest and/or renal angiography with embolization (RAE), has become increasingly popular, especially for low-grade (I–II) and intermediate grade (III) blunt renal injuries (BRI). High-grade renal trauma has traditionally been managed via surgical exploration and published evidence is unclear about the role of NOM for higher grades (IV and V) [4–6]. Over the last few decades, the management of patients with multiple injuries has improved thanks to specialized trauma units and NOM has become the treatment of choice for most renal injuries [7]. Hemodynamic stability is one of the most important criteria to decide between operative and non-operative strategies in renal injuries [8]. As a whole, primary conservative management is associated with a lower rate of nephrectomies, without increasing the immediate or long-term morbidity. The improved staging of injury severity, thanks to advancing radiographic techniques, has enhanced the use of NOM [9]. Furthermore, selective treatment with interventional radiology procedures has eventually reduced the need for surgical interventions in patients with both blunt and penetrating renal injuries [9, 10]. Hemodynamic stability is one of the most important criteria to decide between operative and non-operative strategies in renal injuries [8] Angioembolization has an important role in the management of high-grade BRI [11, 12] but currently, its use remains heterogeneous. The aim of this review is to evaluate the efficacy of angioembolization in blunt renal trauma in current literature, with the aim of understanding the indications and outcomes of RAE in patients with moderate- to high grade renal trauma.

## Methods

PRISMA guidelines [13] were followed, and a literature search was performed using PUBMED, EMBASE, SCOPUS, Web of Science. Articles either published or e-published on angioembolization for BRI between January 2009 and February 2021 were searched. The Mesh terms used for the search were: “renal” (“kidney”); “trauma” (“injury”); “embolization”; “angiography”; (“Renal Angio Embolization” or “RAE”). An additional manual search of EMBASE, as well as bibliographies of each included study, was done to identify studies not covered by the initial search.

### Study eligibility

Studies were selected on the following criteria: (1) patients age > 18 years; (2) studies reporting outcomes of upfront RAE; (3) English language; (4) studies reporting indications, techniques and/or embolic materials. The exclusion criteria were as follows: (1) reviews or editor letters and single case report; (2) non-English language publications; (3) studies involving angioembolization of open injury, penetrating trauma, paediatric patients or iatrogenic injury; and (4) studies with insufficient or unconfirmed information.

Preferred Reporting Items for Systematic Review and Meta-Analysis (PRISMA) guidelines were followed to perform this review. The titles were screened to evaluate whether they met the inclusion criteria. These studies were divided into three different categories: included, probably relevant and excluded. Included and relevant studies were re-evaluated to check eligibility.

### Data extraction and quality assessment

Data including the first author, publication year, number of patients, age, sex, indications, materials, and outcome of interest were extracted by two autonomous reviewers into standardized MS Office Excel. The risk of bias and the methodological quality of eligible studies were assessed using the Newcastle–Ottawa scale [14] which weighs nine items across the domains of selection, comparability and outcome for cohort studies. Each of these three items was assessed and graded (1 or 2 points). In this analysis, studies with NOS scores of 1–3, 4–6, and 7–9, were defined as of low, intermediate, and high quality, respectively. Poor quality studies were not excluded from the review. Two authors independently extracted the data and gave each study a quality assessment; any discordances were resolved through discussion to reach an agreement.

## Results

**Fig. 1:**
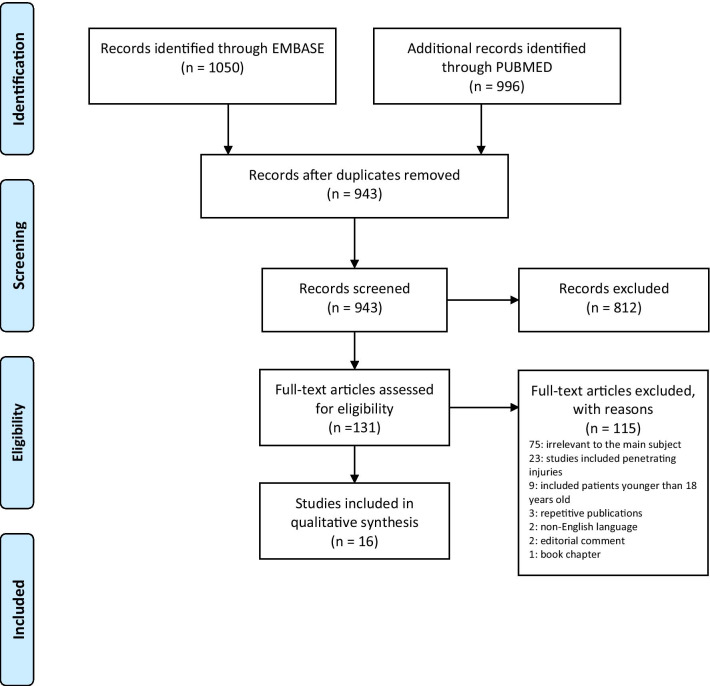
PRISMA flow diagram

Using these search criteria, an initial selection of 1057 articles was considered. After title screening and manual reduplication, we narrowed down to 943 eligible for abstract review. Full-text evaluation for the remaining 131 citations identified by abstract review or by a manual search of the references list was done (Fig. 1). A total of 16 articles that investigated angioembolization in blunt renal injury were included in the study.

### Quality assessment of the included studies

Most studies (68.7%) were graded as intermediate or high quality and full details of the NOS quality appraisal score for each study are summarized in the Additional file 1: Table S1 Quality assessment using the modified Newcastle-Ottawa scale of included cohort studies. Selection and confounding were important sources of error, with only 2 (12.5%) studies attaining the maximum score for selection and no study reaching the maximum score for comparability; 31.2% of cohort designs scored 3/3 for outcome measurement. No prospective studies were available in the current literature.

### Success rate

The clinical success was defined as the absence of repeat RAE, salvage nephrectomy or death for each patient. All 412 patients identified in this review underwent unilateral RAE achieving clinical success in 354 of them (85%) range 75–100% (Tables 1, 2). Overall, 34 patients (8%) experienced surgical exploration for RAE failure: 31 patients underwent salvage nephrectomy (91%) and 3 patients underwent partial nephrectomy (9%). A second embolization, after the failure of the first attempt, was performed in 17 patients and was effective in all of them. The reason for RAE repetition was presence of pseudoaneurysm in 7 cases, recurrence of bleeding in 7 cases, persistent gross haematuria in 3 cases and arteriovenous fistula occurrence in one case, with an overall success rate of 100%. Moreover, 2 patients required subsequent angioplasty after [15] embolization to control arterial bleeding and 4 patients required a more proximal vessel embolization to stop angiographic extravasation [12, 16]. Overall, 404 patients with moderate to high grade renal injuries were included (Table 2): 97 presented with grade III renal trauma (23%); 225 with grade IV (55%); and 82 with grade V (20%). RAE was successful in 92% of cases (294/322) with grade III-IV renal trauma and 76% (63/82) of cases with grade V trauma (76%). Regarding haemodynamic status, success rate was achieved in 312/346 (90%) of stable patients, and only in 42/66 (63%) of unstable patients.

**Table 1:** Summary of patient’s characteristics and clinical outcomes

Year	References	Number of patients	AAST II	AAST III	AAST IV	AAST V	1st embolization success rate (%)	2nd embolization success rate	Salvage Nephrectomy	Perioperative mortality
2009	Brewer [24]	9	0	0	0	9	100	Not performed	RAE successful	12% (1)
2010	Fu [20]	14	0	2	11	1	85	100%	RAE successful	0%
2010	Menaker [36]	22	2	2	17	1	73	Not performed	27% (6)*	0%
2010	Stewart [6]	2	0	0	0	2	100	Not performed	RAE successful	0%
2011	Charbit [19]	10	0	1	4	5	90	Not performed	10% (1)	0%
2012	Van Der Vlies [39]	6	0	1	2	3	100	Not performed	16% (1)	0%
2013	Van de Wilden [12]	25	0	0	20	5	92	100%	RAE successful	0%
2014	Saour [18]	10	0	1	4	5	90	Not performed	10% (1)	0%
2014	Rao [30]	16	0	12	3	1	93	Not performed	7% (1)	6% (1)
2015	Vozianov [16]	20	2	11	7	0	90	100%	RAE successful	0%
2018	Yanagi [21]	17	0	0	17	0	100	Not performed	RAE successful	17% (3)
2019	Pretorius [37]	7	0	3	4	0	85	100%	RAE successful	0%
2020	Chen [38]	7	4	0	3	0	100	Not performed	RAE successful	12% (1)
2020	Desai [17]	15	0	1	12	2	86	Not performed	13% (2)	0%
2020	Xu [15]	62	0	28	20	14	95	Not performed	1.6% (1)	1.6% (1)
2021	Baboudjian [23]	170	0	35	101	34	77	100%	12% (22)	3.5% (6)

*Menaker performed 3 partial nephrectomies

**Table 2:** Outcomes of RAE sorted by hemodynamic status (a) and by AAST category (b)

Patients’ characteristics	Number of patients	RAE Success	RAE Failure
(a) Hemodynamic status			
Overall	412 (100%)	354 (85%)	58 (15%)
Hemodynamic stable patients	346 (85%)	312 (90%)	34 (9%)
Hemodynamic unstable patients	66 (15%)	42 (63%)	24 (37%)
(b) AAST category			
Overall	412 (100%)	354 (85%)	58 (15%)
Grade V	82 (19%)	63 (76%)	19 (24%)
Grade III–IV	322 (77%)	294 (92%)	28 (8%)
Grade II	8 (2%)	8 (100%)	0

Overall, thirteen patients deceased (3%). Seven were hemodynamic unstable patients and died for causes related to the trauma: 4 deaths occurred within 24 h after RAE for uncontrolled bleeding, 1 patient died after surgery and 2 patients died for acute circulation failure despite a successful RAE. The other six patients died during hospitalization for other causes.

Complications were poorly reported. Urinoma was diagnosed as an early complication in 3 patients all successfully managed with ureteral stenting. Four retroperitoneal abscesses were reported and successfully drained. Moreover, two atrophic kidneys were reported as long-term complications by Desai [17]. No patient required dialytic treatment after discharge. Renal function outcome was reported in 3 studies. Xu [15] stated that Grade 3 and 4 lesions did not have a significant decrease in renal function while grade 5 treated with RAE had a significant decrease in glomerular filtration rate (78.8 ± 11.0 vs 72.5 ± 5.6 after RAE). Vozianov [16] reported 2 patients (10%) with a significant drop of renal function at 3 months follow up. Saour [18] reported no significant decrease of renal function after embolization. A brief analysis was performed excluding the lowest quality studies based on risk score ≤ 3. The sub analysis showed an overall success rate of 85% (281/334), success rate in unstable patient of 68% (39/57) while surgical treatment was required in 32 patients (10%).

### Indications for RAE

The most common indication for RAE was contrast extravasation in stable patients with kidney trauma III to V grade. CT criteria, such as absence of major visceral injuries, perirenal hematoma rim distance, discontinuity of Gerota’s fascia, or intravascular contrast extravasation were used to select patients for RAE [12, 19, 20]. Failure of conservative management due to unresponsive to fluid resuscitation hypotension or persistent gross haematuria represent further indication [16, 21–23].

Non operative management with angioembolization was described in hemodynamically unstable patients with high-grade renal injuries, [6, 15, 23, 24] without evidence of other intra-abdominal injuries requiring surgical exploration. Factors predictive of RAE failure were evaluated in one manuscript [23] which reported as significant predictors of RAE failure gross haematuria, hemodynamic instability, grade V trauma, and urinary extravasation.

### Technique of RAE

*Pre-procedure diagnostic work-up* All patients were primarily screened with CT scan to identify the bleeding site. Angiography was performed prior to embolization to define the precise site of bleeding and the actual possibility to perform angioembolization.

*Timing* All patients underwent RAE within 24–48 h of hospital admission in an urgency/emergency setting.

*Route* Most RAE were performed through femoral approach, using 4–6 F sized arterial sheaths [25]. Radial or brachial approach was rare but necessary in case of iliac artery occlusion or unusual anatomic conditions [26].

*Selective catheterization* Selective embolization was performed on angiographic findings, after comparing CT and the arteriogram findings. Comparison between CT and arteriogram allowed the choice between selective embolization or embolization of the proximal branches of the renal artery [6, 19, 24].

*Embolizing agents* the selection mostly depends on interventional radiologists’ preference and diameter of the bleeding vessel. The most popular material was coils (Table 3).

**Table 3:** Embolizing materials

Year	Author	Number of patients	Gelfoam	Pva	Microcoils	NBCA	Microcoils + Gelfoam	Success rate (%)
2009	Brewer [24]	9	44% (4)	0	66% (5)	0	0	100
2010	Stewart [6]	10	0	0	100% (10)	0	0	100
2011	Charbit [19]	10	40% (4)	0	60% (6)	0	0	90
2015	Vozianov [16]	20	30% (6)	70% (14)	0	0	0	90
2018	Yanagi [21]	17	47% (8)	0	11% (2)	5% (1)	35% (6)	100
2020	Desai [17]	15	0	0	86% (13)	0	0	85

Embolizing materials were selected according to radiologist’s preference and diameter of embolized vessel

## Discussion

We present the first systematic review conducted to assess the management and outcomes of RAE in blunt renal injuries. The current analysis suggests that urological outcomes are overall excellent in stable, moderate to high-grade renal trauma without concomitant major visceral injuries. Our findings are consistent with latest guidelines since hemodynamic stability is the main criterion for non-operative management of kidney trauma [27–31]. Angioembolization has a key role in the non-operative management, but no validated criteria to predict RAE efficacy are available in the current literature and its practice remains heterogeneous in blunt renal injuries [23, 32]. We analysed contemporary published articles of patients undergoing RAE for blunt renal trauma. Overall, among the 412 patients with kidney trauma treated with angioembolization, 354 patients (85%) achieved clinical success and avoided further treatments. These results reinforce the current management strategies for renal trauma. A large proportion of high-grade renal trauma could be safely managed with RAE. Moreover, RAE is considered as the first choice for hemodynamically stable patients, achieving an overall 90% success rate (including patients with grade V renal trauma). Conversely, the indications for embolization therapy in grade V are still debated since those injuries are uncommon and most studies lack an appropriate sample size to create statistical significance and deliver valid recommendations. In our review, the success rate for grade V is 73%, but the lack of categorization into renal pedicle injury versus shattered kidney may lead to a misinterpretation. Even if we could assume that RAE has a realistic chance of success in a shattered kidney with extravasations from secondary kidney artery branches, the success rate might be very low if the main renal artery is injured, and the embolization would not be selective. Unfortunately, the patient's selection was not based on a shared protocol, but on the clinical judgment of the trauma-surgeon and radiologist. Furthermore, these decisions could not be retrieved from the trauma registry or electronic medical record, resulting in a selection bias. Baboudijan et al. [23], the largest study to date, retrospectively analysed 170 patients with moderate- to high-grade renal trauma treated with upfront RAE. The authors found that RAE treatment was successful in 131 patients (77%). Subgroup analysis showed RAE efficacy of 64.7% for grade V trauma, 59.6% for patients with hemodynamic instability and 45% for patients with both grade V trauma and hemodynamic instability. Contrarywise*,* the decision to perform angioembolization or surgery is usually based on the presence of concomitant injuries, the hemodynamic status and the grade of the lesion. Overall, in our review, RAE was reported successful in 42 hemodynamically unstable patients with a 63% success rate. Unfortunately, the lack of a control group managed by surgery, prevented drawing strong conclusions around the role of RAE in hemodynamic unstable patients. Hemodynamic instability was defined by Brewer [24] as not responding to Advanced Trauma Life Support protocol, (i.e. presenting with or experiencing the development of hypotension, tachycardia or acidosis, and not responding to 2 l crystalloid and initial blood resuscitation). However, other authors did not define the hemodynamic instability, leading to a possible selection bias. According to Loggers’ review [33], in the current literature there is no consensus on the definition of hemodynamic stability in blunt trauma. Moreover, all patients who died for causes related to kidney trauma were unstable at presentation; therefore, despite the encouraging success rate, this data is still not robust enough to support this treatment as a standard of care.

Overall, only 8 patients (2%) underwent RAE with a grade II renal injury. As a whole, grade I-II are the most frequent types of blunt trauma and usually benefit from a truly conservative approach without embolization. These patients should not be treated routinely with RAE, owing to the risk of inducing unnecessary renal infarctions and overtreatment [34]. In this review, we noted that second RAE or salvage nephrectomy after RAE failure were associated with a high success rate. Both procedures appear to be safe since only one patient (1/33) died after salvage nephrectomy, and none died after the second attempt of RAE. Moreover, three patients underwent RAE before laparotomy for other purposes. These results imply that a first attempt of angioembolization would not prevent the success of a secondary salvage nephrectomy or repeat RAE. The sub-analysis performed without the lowest quality studies showed very similar results to the overall analysis, reinforcing the overall results of this review.

Only one study [23] analysed predictive factors of angioembolization failure. Persistent gross haematuria, hemodynamic instability, and active urine extravasation were associated with RAE failure. Finally, numerous general indications among different embolizing agents have been described (Table 3) [35]; however, the failure rate could not be related to the type of materials.

There are several limitations in this review that should be acknowledged. First of all, a meta-analytic approach was not performed due to the heterogeneity of the studies and the lack of prospective ones. Moreover, retrieved data did not allow us to perform a comprehensive comparative evaluation of RAE vs. observation vs. surgery. Indeed, all studies except one [15] lacked a control group with similar clinical presentation of trauma but managed with either surgery or observation, preventing any solid conclusion on the relative role of RAE. Likewise, very few data on complication were available, and if available still were hardly comparable due to different measurements (i.e., eGFR vs serum creatinine) or different timing (pressure within 24 h of RAE vs at 1–3 months of follow up).

## Conclusions

The use of angioembolization is very limited in low grade blunt renal trauma, where truly conservative management with active monitoring are optimal therapies in the vast majority of cases. Instead, an indication is in stable patients with grade III-IV blunt renal trauma which are likely to benefit from RAE with a very high success rate. Based on very recent studies, the use of embolization is expanding to unstable haemodynamic and grade V renal trauma but to date they represent a contra-indication in most cases. The results in these subgroups are promising, but not entirely reliable since the relevant risk of failure and the significant confounders present in the studies. Multi institutional collaborative research with control group is needed to better assess the relative role of RAE in these subgroups of patients. It is important to remind that surgical management is needed and remains a fundamental component of the trauma surgeon's armamentarium.

## Supplementary Information

**Additional file 1**. Quality assessment using the modified Newcastle-Ottawa scale of included cohort

## Data Availability

All data generated or analysed during this study are included in this published article and its Additional file 1.

## Abbreviations

RAE: Renal angioembolization
NOM: Non operative management
BRI: Blunt renal injuries
CT: Computed tomography
