# Supplementary material for: The role of angioembolization in the management of blunt renal injuries: a systematic review
Source: BMC Urol. 2021 Aug 6;21:104. doi: 10.1186/s12894-021-00873-w (PMC8344199; doi:10.1186/s12894-021-00873-w)
Supplement: Supplementary file 1 — Additional file 1. Quality assessment using the modified Newcastle-Ottawa scale of included cohort [file 12894_2021_873_MOESM1_ESM.docx]

| **Year** | **Author** | **Design** | **Selection** | | | | **Comparability** | **Outcome** | | | **Points** | **Quality of Studies** |
| --- | --- | --- | --- | --- | --- | --- | --- | --- | --- | --- | --- | --- |
|  |  |  | 1 | 2 | 3 | 4 | 5 | 6 | 7 | 8 |  |  |
| **2009** | **Brewer** [24] | Retrospective | - | - | * | * | - | - | - | - | 2 | low |
| **2015** | **Vozianov** [16] | Retrospective | - | - | * | - | - | * | - | - | 2 | low |
| **2019** | **Pretorius** [38] | Retrospective | - | - | * | - | - | - | - | * | 2 | low |
| **2013** | **Van de Wilden** [12] | Retrospective | - | * | * | - | - | * | - | - | 3 | low |
| **2018** | **Yanagi** [21] | Retrospective | * | * | * | * | - | - | * | * | 3 | low |
| **2014** | **Rao** [30] | Retrospective | * | * | * | - | - | - | - | * | 4 | intermediate |
| **2010** | **Menaker** [36] | Retrospective | * | - | * | - | - | * | * | - | 4 | intermediate |
| **2010** | **Stewart** [6] | Retrospective | * | - | * | - | - | * | * | * | 5 | intermediate |
| **2011** | **Charbit** [19] | Retrospective | * | - | * | * | - | - | * | * | 5 | intermediate |
| **2012** | **Van Der Vlies** [37] | Retrospective | * | * | * | - | - | - | * | * | 5 | intermediate |
| **2020** | **Chen** [39] | Retrospective | * | * | * | - | * | * | * | * | 5 | intermediate |
| **2014** | **Saour** [18] | Retrospective | * | * | * | - | * | * | - | * | 6 | intermediate |
| **2010** | **Fu** [20] | Retrospective | * | - | * | * | - | * | * | * | 6 | intermediate |
| **2020** | **Desai** [17] | Retrospective | * | * | * | * | - | - | * | * | 6 | intermediate |
| **2021** | **Baboudjian** [23] | Retrospective | - | * | * | * | * | * | * | * | 7 | high |
| **2020** | **Xu** [15] | Retrospective | * | * | * | * | * | * | * | * | 8 | high |

**Supplement Table 1**

Quality assessment using the modified Newcastle-Ottawa scale of included cohort studies in systematic review of angioembolization in blunt renal injuries
